# A modified Delphi process to identify process of care indicators for the identification, prevention and management of acute kidney injury after major surgery

**DOI:** 10.1186/s40697-015-0047-8

**Published:** 2015-04-09

**Authors:** Matthew T James, Neesh Pannu, Rebecca Barry, Divya Karsanji, Marcello Tonelli, Brenda R Hemmelgarn, Braden J Manns, Sean M Bagshaw, H Tom Stelfox, Elijah Dixon

**Affiliations:** Department of Medicine, University of Calgary, Calgary, Canada; Department of Community Health Sciences, University of Calgary, Calgary, Canada; Department of Medicine, University of Alberta, Edmonton, Canada; Department of Critical Care, University of Alberta, Edmonton, Canada; Department of Critical Care, University of Calgary, Calgary, Canada; Department of Surgery, University of Calgary, Calgary, Canada; Division of Nephrology, Departments of Medicine and Community Health Sciences, University of Calgary, Calgary, T2N 2 T9 AB Canada

**Keywords:** Acute kidney injury, Surgery, Quality indicators

## Abstract

**Background:**

The outcomes of acute kidney injury (AKI) are well appreciated. However, valid indicators of high quality processes of care for AKI after major surgery are lacking.

**Objectives:**

To identify indicators of high quality processes of care related to AKI prevention, identification, and management after major surgery.

**Design:**

A three stage modified Delphi process.

**Setting:**

The study was conducted in Alberta, Canada using an online format.

**Participants:**

A panel of care providers from surgery, critical care, and nephrology.

**Measurements:**

The degree of validity of candidate indicators were rated by panelists on a 7-point Likert scale that ranged from “strongly disagree” to “strongly agree”.

**Methods:**

A focused literature review was performed to identify candidate indicators. A modified Delphi process, with three rounds, was used to obtain expert consensus on the validity of potential process of care quality indicators.

**Results:**

Thirty-three physicians participated (6 from surgery, 10 from critical care, and 17 from nephrology). A list of 58 potential process of care quality indicators for AKI after surgery was generated including 28 indicators from the initial literature review and 30 indicators suggested by panelists. Following the third round of questioning, 40 process of care indicators were identified with a high level of agreement for face validity; 16 of these reached high consensus among all panelists.

**Limitations:**

The consensus of panelists from Alberta, Canada may not be generalizable to other settings. The modified Delphi process did not focus on the feasibility of measuring these process indicators.

**Conclusions:**

These indicators can be used to measure and improve the quality of care for AKI after major surgery.

**Electronic supplementary material:**

The online version of this article (doi:10.1186/s40697-015-0047-8) contains supplementary material, which is available to authorized users.

## Abrégé

**Contexte:**

Les répercussions engendrées par l’insuffisance rénale aiguë (IRA) sont bien connues. Cependant, il n’existe toujours pas d’indicateurs de la qualité du processus de soin de l’IRA valides en phase postopératoire.

**Objectifs de l’Étude:**

Élaborer des indicateurs de la qualité des processus de soin en matière de prévention, d’identification et de prise en charge de l’IRA en phase postopératoire d’une chirurgie majeure.

**Type d’étude:**

Processus Delphi modifié à trois étapes.

**Lieu de l’Étude:**

L’étude a été effectuée en Alberta, Canada, par l’intermédiaire de questionnaires en ligne.

**Participants:**

Panel de professionnels de la santé des milieux suivants : chirurgie, soins intensifs et néphrologie.

**Mesures:**

Les panelistes ont évalué le degré de validité des indicateurs potentiels avec une échelle de Likert à 7 éléments, dont l’étendue se situait entre « fortement en désaccord » (valeur originale : strongly disagree) et « fortement en accord » (valeur originale : strongly agree).

**Méthode:**

Une revue de la littérature ciblée a été effectuée pour faire ressortir les indicateurs pertinents. Une version modifiée du processus Delphi, comprenant trois étapes, a été utilisée dans le but d’obtenir l’avis des experts sur la validité des indicateurs potentiels en matière de qualité des processus de soin.

**Résultats:**

Trente-trois médecins ont participé au panel (6 chirurgiens, 10 intensivistes et 17 néphrologues). Une liste de 58 indicateurs potentiels de la qualité des processus de soin pour l’IRA en période postopératoire a été élaborée; celle-ci comprenait 28 indicateurs qui provenaient de la revue de la littérature et 30 qui avaient été suggérés par les panelistes. Après la troisième ronde de questionnaires, 40 indicateurs des processus de soins avaient été retenus pour leur validité apparente, avec un haut niveau d’accord parmi le panel, et 16 de ces indicateurs avaient atteint un fort consensus.

**Limites de l’Étude:**

Le consensus qui provient du panel formé en Alberta, Canada, n’est peut-être pas généralisable à d’autres contextes. Le processus Delphi modifié ne s’est pas penché sur la faisabilité de la mesure des indicateurs de processus de soin.

**Conclusion:**

Ces indicateurs peuvent être utilisés pour mesurer et améliorer la qualité des soins de l’IRA en période postopératoire d’une chirurgie majeure.

## What was known before

Variability in processes of care for acute kidney injury (AKI) is common. Identification of valid process of care indicators is useful to assess the quality of care, so that it can be measured, monitored, and targeted for improvement.

## What this adds

This study identified 40 process of care quality indicators with high perceived validity, including 16 that reached high consensus among all expert panelists, for the prevention, identification, and management of AKI after major surgery. These indicators can be used to design and evaluate initiatives to improve the care of patients with or at risk of AKI after surgery.

## Background

AKI is a common problem, with an incidence of 4-7% among all hospital admissions [1-4] and 10-30% after major surgery. AKI is associated with morbidity, increased length of hospital stay, progression to end-stage renal disease (ESRD), high healthcare costs, and death [5-10]. The utilization of dialysis for AKI after major surgery has increased more than 3-fold over the last 10 years in Canada, contributing to longer hospital stays and higher costs of care [11].

While the important outcomes of AKI have been broadly recognized, a lack of consensus remains about high quality processes of care that are important for prevention and management of AKI [12,13]. Variability in processes of care for AKI is reported to be common, despite calls for initiatives to improve the quality of care for patients with or at high risk of AKI [14-16]. Identification of valid process of care indicators for AKI after major surgery would be useful to assess the quality of this care, so that it can be measured, monitored and targeted for improvement [17-20]. Several recently published clinical practice guidelines provide recommendations for AKI prevention, identification and treatment; however, few aspects of AKI prevention and management have been evaluated in rigorously conducted empirical studies in the perioperative setting, and so quality indicators for AKI prevention and care must be selected partly based on the opinions and experience of clinicians with knowledge in this area [21,22].

To inform the development of future initiatives to improve care for AKI after major surgery, we undertook a 3-stage, modified Delphi process with care providers from surgery, critical care, and nephrology in Alberta, Canada. Our aim was to achieve consensus among experts about the most important processes of care for prevention, recognition and management of AKI after major surgery.

## Methods

This modified process was performed according to a pre-specified protocol and adhered to published recommendations for reporting [23]. Approval of the study was obtained from the Conjoint Health Research Ethics Board of the University of Calgary.

### Panel selection

A mixed-specialty panel of participants was selected based on known clinical expertise in the areas of surgery, critical care, and nephrology. All invited panelists were physicians with experience caring for patients with or at risk of AKI in Alberta. Participants were approached with an introductory email including a letter of invitation outlining the method and reason their participation in the whole process was important. A subpanel of 6 researchers, including 3 with expertise in the Delphi technique, was created to provide input to refine each round of questioning.

### Literature search

As an initial step, a focused literature search was performed to identify guideline recommendations and potential process of care indicators for the prevention, identification, and general management of AKI after major surgical and contrast imaging procedures. The literature search involved searches of Medline, EMBASE, and the Cochrane library without date limits or language restriction and was completed in the last week of April 2013. The literature search was based on search terms related to three themes: AKI, clinical guidelines, and quality indicators (Additional file 1). Grey literature was also searched using the Google Search engine as well as unpublished internet reports, guidelines, and society reports on AKI management. Two reviewers examined the identified literature, extracted pertinent guideline statements from the search yield, and grouped the extracted statements about process indicators according to the relevant phase of AKI care (prevention, identification, and management).

### List of potential indicators

A list of potential process of care indicators was generated from the literature search. The initial list was modified by the 6 member subpanel to reduce redundancy by combining common process indicators where possible and limiting potential indicators to actionable items. For the purposes of our work, guideline statements or potential indicators focusing on delivery of renal replacement therapy for AKI were excluded.

### Overview of modified Delphi process

Three rounds of questions were distributed via Internet-based questionnaires to all participants between September 2013 and May 2014. Panelists provided consent to participate in the entire modified Delphi process on an online form distributed with the first round of questions. Reminder emails were sent to non-respondents at two and four weeks after distribution of each round. Panelists were given the option of using mailed questionnaires; however, all participants chose to respond using the Internet.

### Round 1

Panelists were emailed a user-unique link to a questionnaire developed using the online software FluidSurveys (http://fluidsurveys.com). Panelists were first asked about their number of years in clinical practice, their primary specialty, and the number of patients with AKI they typically care for each year. The refined list of AKI clinical process of care indicators developed from the literature search was presented to panelists, grouped according to statements for perioperative AKI prevention/monitoring, contrast-induced AKI prevention, monitoring/management of AKI, and specialist consultation for AKI. Panelists were asked to respond to the validity of each statement with “agree”, “disagree”, or “unsure”. Open-ended questions were also provided, asking panelists to list other clinical processes of care that they felt were important for each of these aspects of AKI. Panelists were also asked to provide criteria that they felt should prompt a consult with a specialist (critical care physicians or nephrologist) for a patient with AKI.

### Round 2

The second round of the survey was based on a refined list of additional process of care indicators. The refined list was developed from responses to the open-ended questions collected in Round 1. Panelists were again asked to respond to the validity of each candidate indicator with “agree”, “disagree”, or “unsure”. No open-ended questions were provided in this round.

### Round 3

The third round of the survey included all process of care indicators provided in Rounds 1 and Round 2. In this round, the percentage of panelists that responded “agree” to each candidate indicator in the previous rounds was provided. Panelists were asked to respond to the degree of validity of each candidate indicator using a 7-point Likert scale that ranged from “strongly disagree” to “strongly agree”.

### Analysis

Results were tabulated at the completion of each round and entered into an Excel worksheet. The percentage agreement was calculated for the response to each question after Rounds 1 and 2 (Appendix 1). Following the completion of Round 3, mean (95% confidence interval) responses on the 7-point Likert scale were calculated for each question. Candidate indicators with mean scores of 0–3 were categorized as of low perceived validity, 4–5 as moderate perceived validity, and 6–7 as representing high perceived validity. Process indicators with a mean score greater than 6 were identified as having a high degree of validity and included in a final list of valid indicators. Those indicators for which no panelists assigned a score less than six were identified as high consensus indicators.

## Results

A total of 51 clinicians were invited as potential panelists, and 33 (64.70%) agreed to participate. The characteristics of the panelists are shown in Table 1. There were 6 panelists (18.2%) from surgery, 10 (30.3%) from critical care, and 17 (51.5%) from nephrology. The range of post-training clinical practice among panelists ranged from less than 5 to more than 30 years. Most reported that they cared for more than 60 patients with AKI each year.

**Table 1:** **Characteristics of panelists (n = 33)**

	**Number (%)**
Years in Clinical Practice	
<5	6 (18)
5-9	9 (27)
10-19	10 (30)
20-29	7 (21)
30-39	1 (3)
>40	0 (0)
Primary Area of Specialty	
Surgery	6 (18)
Nephrology	17 (52)
Critical Care	10 (30)
Approximate number of patients a year cared for with acute kidney injury	
<20	1 (3)
20-39	9 (27)
45-59	4 (12)
>60	19 (58)

**Figure 1:**
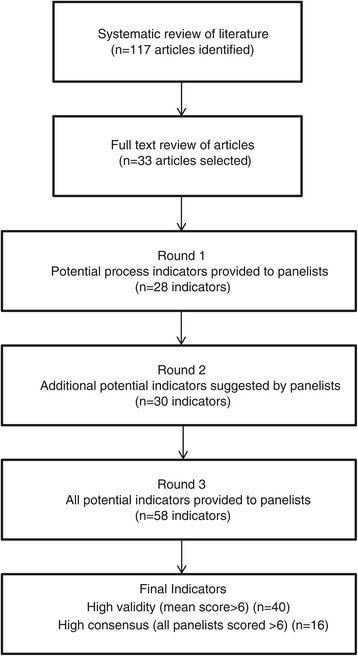
**Progress through steps of the literature search and modified Delphi procedure to identify process of care indicators for the identification, prevention and management of acute kidney injury after major surgery.**

**Figure 2:**
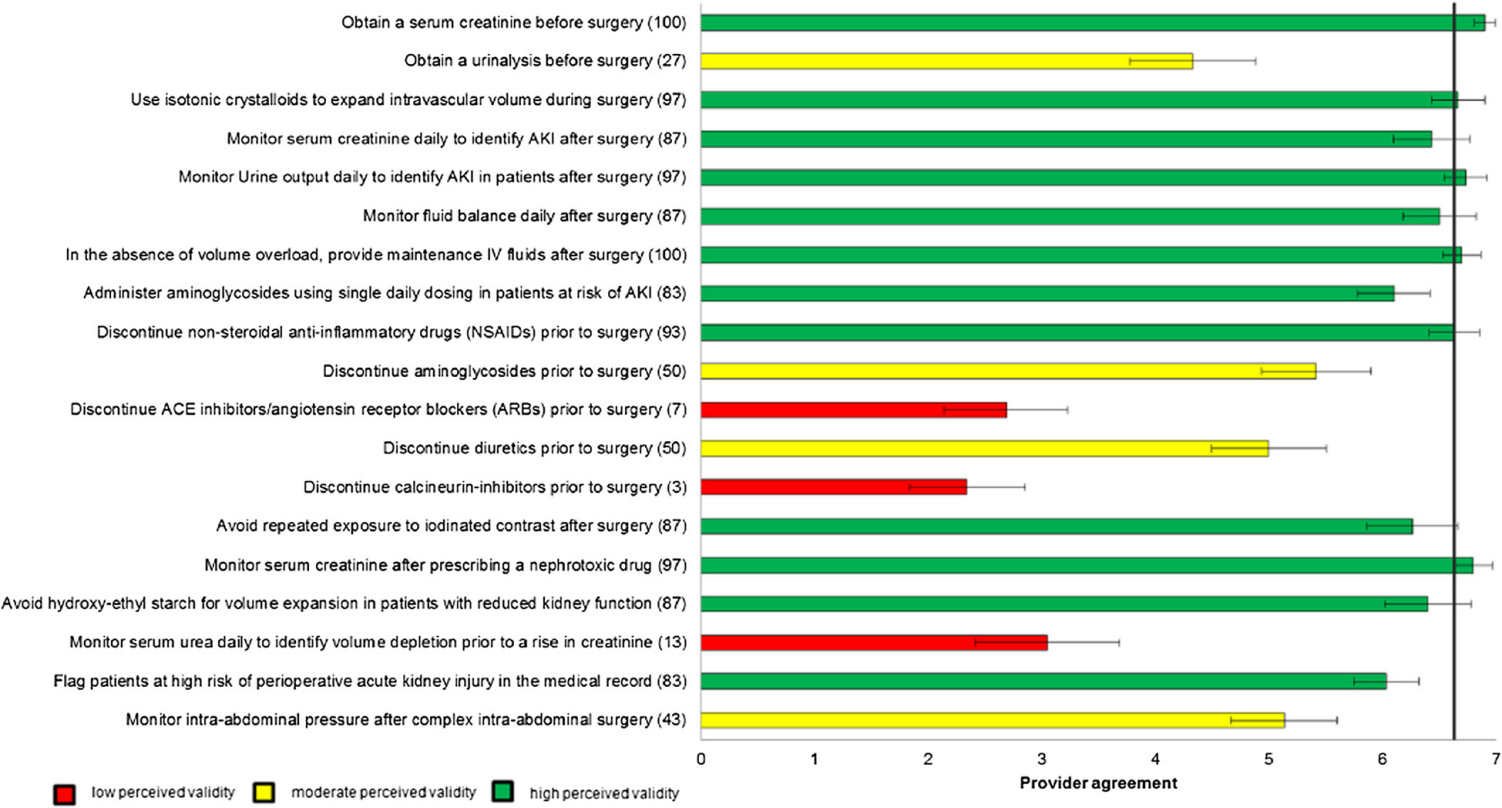
**Perceived Validity of Candidate Process of Care Quality Indicators for Prevention and Early Identification of Acute Kidney Injury after Major Surgery.** The bars represent the mean scores with 95% confidence intervals. The number in brackets following each indicator represents the percentage of participants that stated they agreed with the indicator in the initial round of questioning, and was presented to participants in the final round. The horizontal line represents the median score for all indicators.

**Figure 3:**
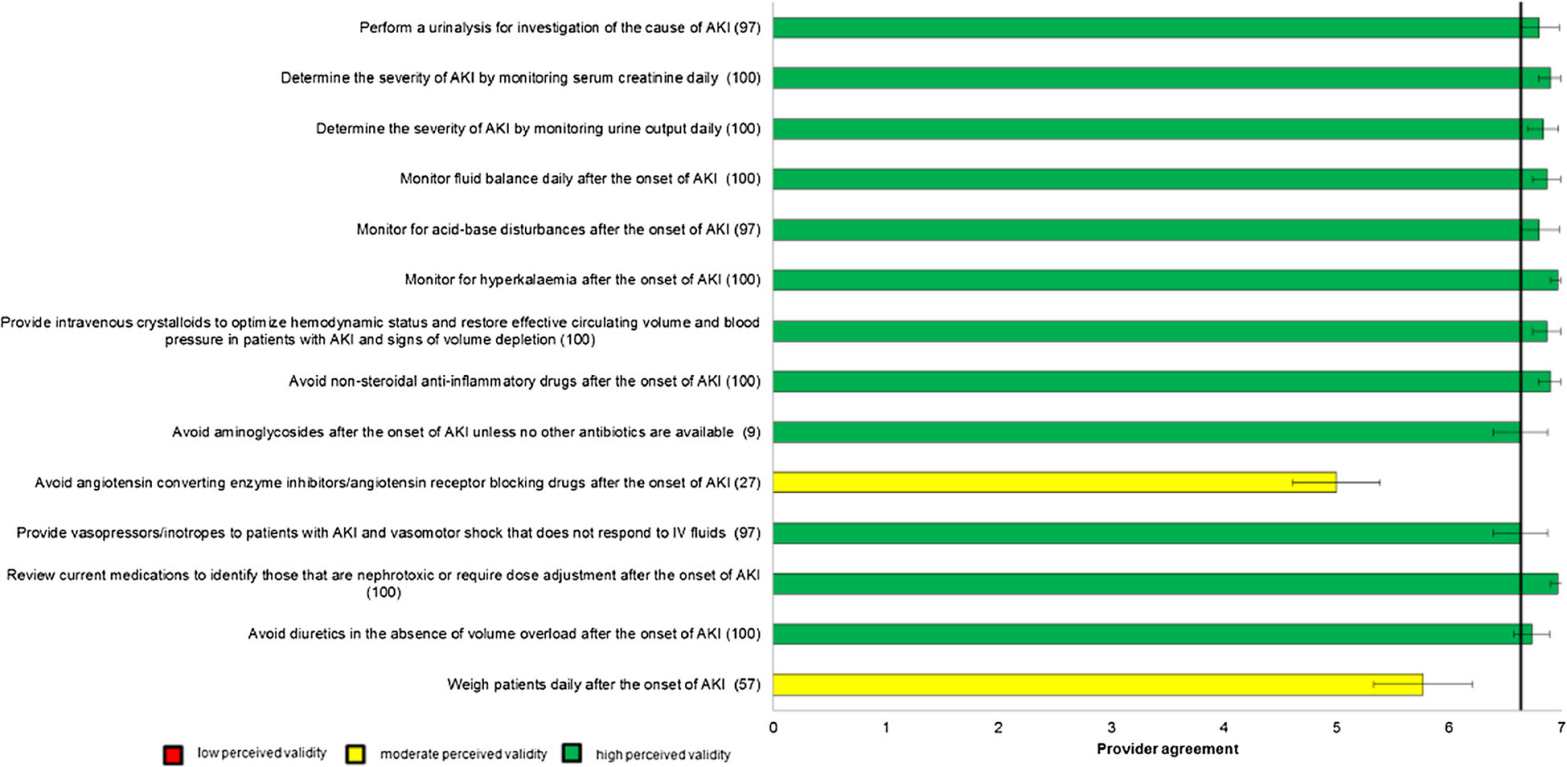
**Perceived Validity of Candidate Process of Care Quality Indicators for Early Management of Acute Kidney Injury after Major Surgery.** The bars represent the mean scores with 95% confidence intervals. The number in brackets following each indicator represents the percentage of participants that stated they agreed with the indicator in the initial round of questioning, and was presented to participants in the final round. The horizontal line represents the median score for all indicators.

**Figure 4:**
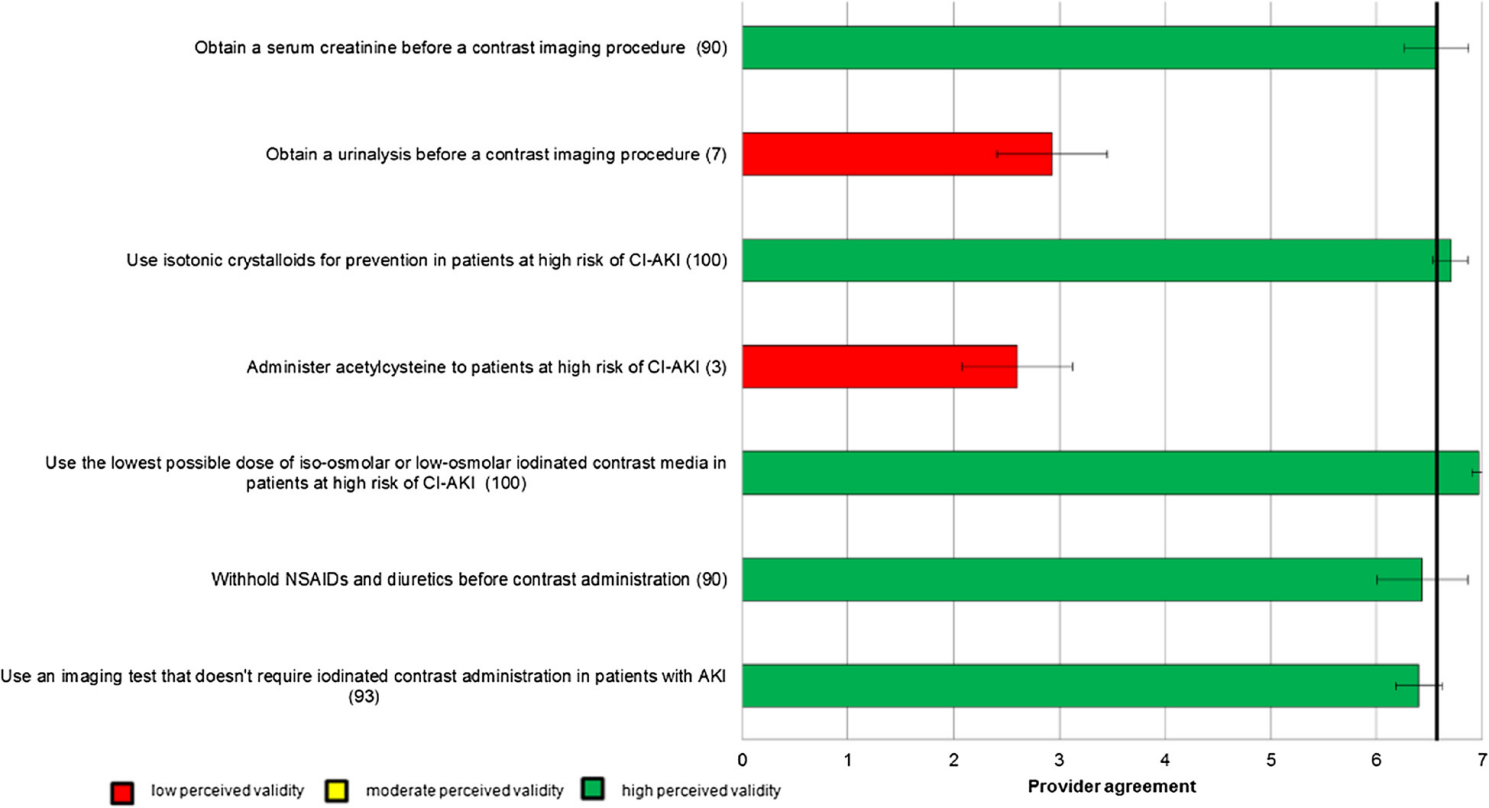
**Perceived Validity of Candidate Process of Care Quality Indicators for Prevention of Contrast-induced AKI.** The bars represent the mean scores with 95% confidence intervals. The number in brackets following each indicator represents the percentage of participants that stated they agreed with the indicator in the initial round of questioning, and was presented to participants in the final round. The horizontal line represents the median score for all indicators.

**Figure 5:**
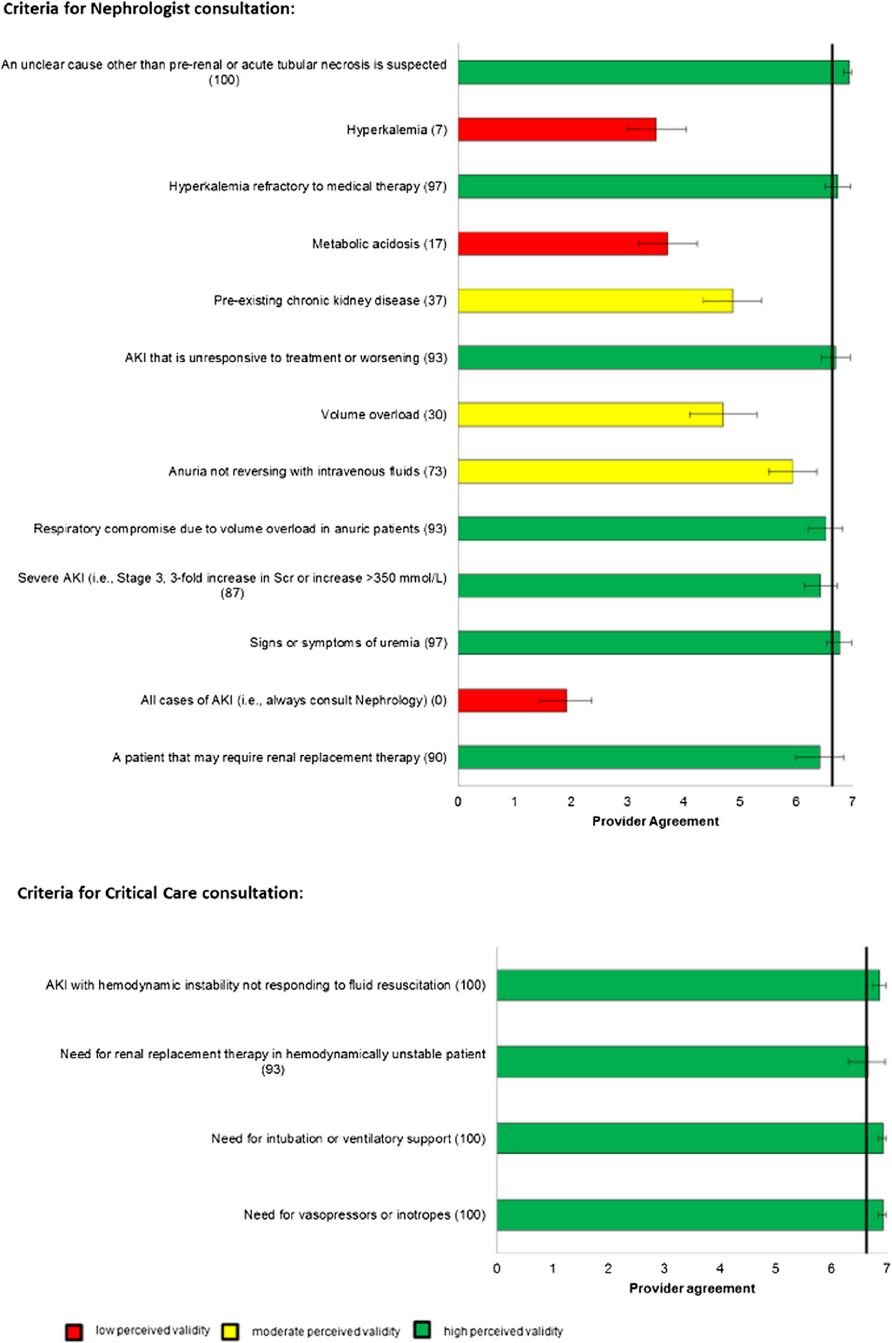
**Perceived Validity of Candidate Process of Care Quality Indicators for Specialist Consultation for Acute Kidney Injury.** The bars represent the mean scores with 95% confidence intervals. The number in brackets following each indicator represents the percentage of participants that stated they agreed with the indicator in the initial round of questioning, and was presented to participants in the final round. The horizontal line represents the median score for all indicators.

In total, 31 (97%) panelists who agreed to participate responded to Round 1. Panelists provided responses to 28 process of care indicators identified from the literature review, and provided open ended responses that resulted in 30 additional potential process of care indicators for further evaluation (Figure 1). In Round 2, 27 (82%) panelists responded to the questionnaire. A summary of responses to the full 58 process of care statements provided in Rounds 1 and 2 of the Delphi process is provided in brackets following the text statements in Figures 2-5.

In total, 30 (91%) panelists responded to the questionnaire in Round 3. Mean (95% confidence intervals) scores for each of the process of care indicators are shown graphically in Figures 2. Forty process of care indicators had a mean score greater than 6, indicating high agreement for validity (Table 2); 12 were for prevention and early Identification of AKI after major surgery, 12 were for early management of AKI after major surgery, 5 were for prevention of contrast-induced AKI after major surgery, and 11 were criteria for nephrologist or critical care consultation in AKI. Of these 40 process of care quality indicators with high perceived validity, 22 (55%) were indicators originally identified from the literature review and 18 (45%) were indicators suggested by panelists. Of the 40 indicators, 16 (40%) were scored greater than 6 by all participants consistent with the definition of high consensus indicators.

**Table 2:** Process of care quality indicators identified as having a high degree of validity in the prevention, identification, and management of acute kidney injury (AKI) after major surgery*

	**Number (%) who scored indicator ≥6/7** ^**†**^
***Prevention and Early Identification of AKI after major surgery***	
Obtain a serum creatinine before surgery	**30 (100)**
Use isotonic crystalloids to expand intravascular volume during surgery	29 (97)
Monitor serum creatinine daily to identify AKI after surgery	26 (87)
Monitor urine output daily to identify AKI after surgery	29 (97)
Monitor fluid balance daily after surgery	26 (87)
In the absence of volume overload, provide maintenance IV fluids after surgery	**30 (100)**
Administer aminoglycosides using single daily dosing in patients at risk of AKI	25 (83)
Discontinue non-steroidal anti-inflammatory drugs prior to surgery	28 (93)
Avoid repeated exposure to iodinated contrast after surgery	26 (87)
Monitor serum creatinine after prescribing a nephrotoxic drug	29 (97)
Avoid hydroxy-ethyl starch for volume expansion in patients with reduced kidney function^‡^	26 (87)
Flag patients at high risk of perioperative acute kidney injury in the medical record	28 (93)
***Early Management of AKI After major surgery:***	
Perform a urinalysis for investigation of the cause of AKI	29 (97)
Determine the severity of AKI by monitoring serum creatinine daily after the onset of AKI	**30 (100)**
Determine the severity of AKI by monitoring urine output daily after the onset of AKI	**30 (100)**
Monitor fluid balance daily after the onset of AKI	**30 (100)**
Monitor for acid–base disturbances after the onset of AKI	29 (97)
Monitor for hyperkalemia after the onset of AKI	**30 (100)**
Provide intravenous crystalloids to optimize hemodynamic status and restore effective circulating volume and blood pressure in patients with AKI and signs of volume depletion	**30 (100)**
Avoid non-steroidal anti-inflammatory drugs after the onset of AKI	**30 (100)**
Avoid aminoglycosides after the onset of AKI unless no other antibiotics are available	27 (90)
Provide vasopressors/inotropes to patients with AKI and vasomotor shock that does not respond to IV fluids	29 (97)
Review current medications to identify those that are nephrotoxic or require dose adjustment after the onset of AKI	**30 (100)**
Avoid diuretics in the absence of volume overload after the onset of AKI	**30 (100)**
***Recommendations for Prevention of Contrast-induced AKI:***	
Obtain a serum creatinine before a contrast imaging procedure	27 (90)
Use isotonic crystalloids for prevention in patients at high risk of CI-AKI	**30 (100)**
Use the lowest possible dose of iso-osmolar or low-osmolar iodinated contrast media in patients at high risk of CI-AKI	**30 (100)**
Withhold NSAIDs and diuretics before contrast administration	27 (90)
Use an imaging test that doesn’t require iodinated contrast administration in patients with AKI	28 (93)
***Criteria for Nephrologist Consultation:***	
An unclear etiology or cause other than pre-renal or acute tubular necrosis is suspected	**30 (100)**
Hyperkalemia refractory to medical therapy	29 (97)
AKI that is unresponsive to treatment or worsening	28 (93)
Respiratory compromise due to volume overload in anuric patients	28 (93)
Severe AKI (i.e., KDIGO Stage 3, 3-fold increase in serum creatinine or increase in Scr > 350 μmol/L)	26 (87)
Signs or symptoms of uremia	29 (97)
A patient that may require renal replacement therapy	27 (90)
***Criteria for Critical Care Consultation***	
AKI with hemodynamic instability not responding to fluid resuscitation	**30 (100)**
Need for renal replacement therapy in hemodynamically unstable patient	28 (93)
Need for intubation or ventilatory support	**30 (100)**
Need for vasopressors or inotropes	**30 (100)**

*All indicators listed in this table achieved high scores for validity based on a mean score >6 on a 7-point Likert scale.

^†^The number (%) of panelists who scored each indicator ≥ 6 is shown in the column on the right, with the 16 high consensus indicators, for which no panelists assigned a score <6, highlighted in bold.

^‡^Applicable to all patients since hydroxyl-ethyl starches shown to increase the risk of AKI and renal replacement therapy in all populations (Mutter TC, Ruth CA, Dart AB. Hydroxyethyl starch (HES) versus other fluid therapies: effects on kidney function. Cochrane Database Syst Rev. 2013 Jul 23;7).

## Discussion

Using a modified Delphi method involving a panel of Alberta physician specialists, we identified 40 process of care quality indicators with high perceived validity for the prevention, identification, and management of AKI after major surgery. Sixteen of these process indicators were identified by panel members with high consensus.

The process of care quality indicators identified as having high validity by our panelists demonstrate the perceived importance of risk stratification and identification of patients at high risk of AKI as well as clinical and laboratory monitoring for early identification of AKI. Our findings illustrate high agreement for monitoring serum creatinine, urine output, and fluid balance after major surgery, with the addition of laboratory testing for, acidosis, and hyperkalemia following AKI onset. Careful monitoring of these measures may aid early recognition of AKI and, provide opportunities to intervene before severe complications develop [6]. This is consistent with recent guidelines for AKI that recommend routine monitoring of patients at risk of AKI in order to ensure timely recognition of AKI [21,24].

With respect to early management of AKI post surgery, indicators including use of intravenous crystalloids for intravascular volume expansion, withholding non-steroidal anti-inflammatory drugs (NSAIDs), aminoglycosides, and withholding or avoiding diuretics in the absence of volume overload, and reviewing current medications to identify those that are nephrotoxic or require dose adjustment also achieved high agreement by panelists. This is consistent with research suggesting that these medications are modifiable risk factors for AKI [6,7,21,24]. There was also general consensus among panel members that several process of care indicators for prevention of contrast-induced AKI were important. These included indicators to avoid NSAIDs and diuretics prior to contrast exposure and to minimize or avoid the use of intravenous contrast when possible.

There was high agreement on four indicators of need for critical care consultation when AKI was accompanied by circulatory or respiratory failure. However, only one of seven final indicators for nephrology consult reached consensus. This may reflect varying perspectives about the role nephrologists play in management of typical cases of AKI due to pre-renal causes or acute tubular necrosis after surgery [25-27].

These process of care quality indicators may be used in future quality improvement or research studies of perioperative AKI. Researchers could use these indicators as targets for interventions designed to improve the quality of care, or to evaluate changes in process of care with knowledge translation interventions including audit and feedback or organizational interventions. These indicators could also be targeted by formal educational interventions or employed as teaching tools for medical trainees.

Our study has a number of strengths. First, the modified Delphi procedure allows for anonymity of responses from panelists, which may provide a more accurate account of their beliefs surrounding best practices by reducing social desirability bias [28]. This approach minimized the risk of a dominant panelist unduly influencing the panel that can be observed in a less structured setting, such as a focus group, and allowed each panelist to present their opinions with equal weighting, regardless of seniority [29,30]. Second, this Delphi process was conducted via the Internet allowing inclusion of panelists from multiple locations across Alberta to participate. Third, the process allowed participants to re-evaluate their beliefs after receiving feedback on responses from other panelists in the third round. Finally, as candidate indicators were based on published and unpublished literature as well as the opinions of panelists, our results incorporated different forms of evidence to develop a broad range of process indicators.

There are also limitations to our study. First, all panelists were from Alberta Canada, thus external validity may be limited in other health care settings where practices differ. However, the challenges of prevention, early identification and management of AKI following major surgery are likely to be similar across different health care settings. Second, we focused on medical care for AKI, and did not include anesthesiologists who may have provided different perspectives on intraoperative processes of care. Third, we focused on processes of care that could be generalized to all patients after major surgery, rather than features of specific types of surgical procedures themselves (e.g. on-pump versus off-pump cardiac surgery) that may alter the risk of AKI. Finally, this modified Delphi process focused on obtaining input on the face validity but not the feasibility of measuring these process indicators for AKI [23]. Future work is required to determine the practicality and accuracy of measuring these variables in clinical settings.

## Conclusion

In summary, we used an iterative stepwise approach to obtain expert clinician opinions on indicators of process of care for prevention, identification, and management of perioperative AKI. These indicators can be used to design and evaluate initiatives to improve the care of patients with or at risk of AKI after surgery.

## Appendix 1 All process of care indicators

***Prevention and Early Identification of AKI after major surgery (12)***

Obtain a serum creatinine before surgery

Use isotonic crystalloids to expand intravascular volume during surgery

Monitor serum creatinine daily to identify AKI after surgery

Monitor urine output daily to identify AKI after surgery

Monitor fluid balance daily after surgery

In the absence of volume overload, provide maintenance IV fluids after surgery

Administer aminoglycosides using single daily dosing in patients at risk of AKI

Discontinue non-steroidal anti-inflammatory drugs prior to surgery

Avoid repeated exposure to iodinated contrast after surgery

Monitor serum creatinine after prescribing a nephrotoxic drug

Avoid hydroxy-ethyl starch for volume expansion in patients with reduced kidney function

Flag patients at high risk of perioperative acute kidney injury in the medical record

Obtain a urinalysis before surgery

Discontinue aminoglycosides prior to surgery

Discontinue ACE inhibitors/angiotensin receptor clockers (ARBs) prior to surgery

Discontinue diuretics prior to surgery

Discontinue calcineurin-inhibitors prior to surgery

Monitor serum urea daily to identify volume depletion prior to a rise in serum creatinine

Monitor intra-abdominal pressure after complex intra-abdominal surgery

***Early Management of AKI After major surgery (12)***

Perform a urinalysis for investigation of the cause of AKI

Determine the severity of AKI by monitoring serum creatinine daily after the onset of AKI

Determine the severity of AKI by monitoring urine output daily after the onset of AKI

Monitor fluid balance daily after the onset of AKI

Monitor for acid–base disturbances after the onset of AKI

Monitor for hyperkalemia after the onset of AKI

Provide intravenous crystalloids to optimize hemodynamic status and restore effective circulating volume and blood pressure in patients with AKI and signs of volume depletion

Avoid non-steroidal anti-inflammatory drugs after the onset of AKI

Avoid aminoglycosides after the onset of AKI unless no other antibiotics are available

Provide vasopressors/inotropes to patients with AKI and vasomotor shock that does not respond to IV fluids

Review current medications to identify those that are nephrotoxic or require dose adjustment after the onset of AKI

Avoid diuretics in the absence of volume overload after the onset of AKI

Avoid angiotensin converting enzyme inhibotors/angiotensin receptor blocking drugs after the onset of AKI

Weigh patients daily after the onset of AKI

***Recommendations for Prevention of Contrast-induced AKI (5)***

Obtain a serum creatinine before a contrast imaging procedure

Use isotonic crystalloids for prevention in patients at high risk of CI-AKI

Use the lowest possible dose of iso-osmolar or low-osmolar iodinated contrast media in patients at high risk of CI-AKI

Withhold NSAIDs and diuretics before contrast administration

Use an imaging test that doesn’t require iodinated contrast administration in patients with AKI

Obtain a urinalysis before a contrast imaging procedure

Administer acetylcysteine to patients at high risk of CI-AKI

***Criteria for Nephrologist Consultation (7)***

An unclear etiology or cause other than pre-renal or acute tubular necrosis is suspected

Hyperkalemia refractory to medical therapy

AKI that is unresponsive to treatment or worsening

Respiratory compromise due to volume overload in anuric patients

Severe AKI (i.e., KDIGO Stage 3, 3-fold increase in serum creatinine or increase in Scr>350 μmol/L)

Signs or symptoms of uremia

A patient that may require renal replacement therapy

Hyperkalemia

Metabolic acidosis

All cases of AKI (i.e., always consult Nephrology)

Pre-existing kidney disease

Volume overload

Anuria not reversing with intravenous fluid

***Criteria for Critical Care Consultation (4)***

AKI with hemodynamic instability not responding to fluid resuscitation

Need for renal replacement therapy in hemodynamically unstable patient

Need for intubation or ventilatory support

Need for vasopressors or inotropes

## Additional file

Additional file 1:
**Literature search terms.**
