# Supplementary material for: A modified Delphi process to identify process of care indicators for the identification, prevention and management of acute kidney injury after major surgery
Source: Can J Kidney Health Dis. 2015 Apr 9;2:11. doi: 10.1186/s40697-015-0047-8 (PMC4460967; doi:10.1186/s40697-015-0047-8)
Supplement: Additional file 1: — Literature search terms. [file 40697_2015_47_MOESM1_ESM.docx]

**Additional file 1 – Literature Search Terms**

**EMBASE**

1. Acute Kidney Injury.mp. or exp acute kidney failure/

2. exp quality control/ or quality indicator.mp.

3. quality assurance.mp. or exp quality control/

4. performance improvement.mp.

5. quality measure.mp.

6. exp practice guideline/

7. exp practice guideline/ or best practice.mp.

8. audit.mp. or exp medical audit/

9. (8 or 7 or 5 or 4 or 3 or 2) and 1

**Medline**

1. Acute Kidney Injury.mp. or exp Acute Kidney Injury/

2. quality indicator.mp. or exp Quality Indicators, Health Care/

3. exp Quality Control/ or exp Quality Assurance, Health Care/

4. performance improvement.mp.

5. exp "Outcome Assessment (Health Care)"/ or quality measure.mp. or exp "Outcome and Process Assessment (Health Care)"/

6. best practice.mp.

7. audit.mp. or exp Clinical Audit/

8. (7 or 6 or 5 or 4 or 3 or 2) and 1

**COCHRANE Library**

1. Acute Kidney Injury.mp

2. Acute renal failure. mp

3. Quality indicator.mp.

4. Quality assurance.mp.

5. Quality control.mp.

6. Performance improvement.mp.

7. quality measure.mp.

8. best practice.mp.

9. audit.mp.

10. (9 or 8 or 7 or 6 or 5 or 4 or 3) and (1 or 2)
